# Level of Patient Satisfaction with Inpatient Services and Its Determinants: A Study of a Specialized Hospital in Ethiopia

**DOI:** 10.1155/2020/2473469

**Published:** 2020-08-13

**Authors:** Nebsu Asamrew, Abduilhafiz A. Endris, Musse Tadesse

**Affiliations:** ^1^Health Service Quality Directorate, Addis Ababa Regional Health Bureau, Addis Ababa, Ethiopia; ^2^Public Health Emergency Management Center, Ethiopian Public Health Institute, P.O. Box: 1242, Addis Ababa, Ethiopia

## Abstract

**Background:**

The health care industry is undergoing a rapid transformation to meet the ever-increasing needs and demands of its patient population. Level of patients' satisfaction is an important health outcome, which is regarded as a determinant measure for quality of care. This study was performed with the aim of assessing the level of patient satisfaction with inpatient services and its determinants in Black Lion Specialized Hospital, Addis Ababa, Ethiopia.

**Methods:**

A facility-based cross-sectional study was conducted from November 25^th^ to December 20^th^, 2015, using 398 randomly selected patients. Ethical clearance was obtained from the Jimma University research review board, and verbal consent was also received from the study participants during data collection time. A pretested structured interview questionnaire was used to collect data from study participants. The collected data were handled by using SPSS statistical software. Before analysis, relevant explanatory variables were identified using factor analysis with varimax rotation, and bivariate analysis was carried out using linear regression for every independent variable with the outcome variable independently. Explanatory variables scoring *p* value <−0.05 were used for the final model after checking the assumption. Study findings are presented by using tables, graphs, and description.

**Results:**

A total of 398 patients were participated in the study, yielding a response rate of 100%. A total of 46.2% (95% CI: 41.2%–51.1%) patients were satisfied by the services they received in the hospital. Patient and health care provider interaction and general facility amenity-related domains were found to explain 96.4% of the variability in the net overall satisfaction score. Good quality services provided by hospital physicians, availability of laboratory and radiology services, pain management services, and inpatient pharmacy services of the hospital had positive influences. Besides toilet cleanliness, availability of rooms for accommodation and dietary service had significant relation with level of patient satisfaction. Quality of the inpatient pharmacy service had a great influence on satisfaction; a unit increase in it resulted in 2.3 (95% CI: 2.1–2.5) times increment in patient satisfaction level at *p* ≤ 0.001. For final predictors, regression estimates for level of satisfaction moved from very dissatisfied to very satisfied when service improves by a unit.

**Conclusion:**

Overall patients' satisfaction is lower than other studies in the nation. A great opportunity is there to improve patient's satisfaction level if the service quality is improved around the time of patient and health care provider interaction and facility amenity services. Besides, improving the health literacy of service providers and devising a strategy to routinely assess satisfaction level of patients in the facility is critical. On top of this, providing tailored on-the-job training for health care workers in the facility is a crucial step in order to improve their knowledge and skills to render patient-centered quality service to improve their patients' satisfaction. Using a checklist during service delivery may improve client patient interaction and ensure the standard. Facility design dimension can be considered for future research activities.

## 1. Background

### 1.1. Introduction

Health care quality is becoming a global issue, which occasioned the health care industry to undergo a rapid transformation to meet the ever-increasing needs and demands of its patient population [1, 2]. Quality of health services was traditionally based on professional practice standards; however, over the last decade, patient's perception about health care has been predominantly becoming an important indicator for measuring the quality of health care [3]. On top of this, patient satisfaction is becoming a critical component of performance improvement and clinical effectiveness [2, 4–6].

Recent practices in the health system showed that health authorities are considering the patient as the best judge who accurately assesses and provides inputs to help in the overall improvement of quality health care provision through the rectification of the system weaknesses [1, 2, 6]. The regulatory and accreditation mandates imposed by agencies such as the Joint Commission on Accreditation of Healthcare Organizations (JCAHO) and the National Committee on Quality Assurance (NCQA) showed that quantifiable patient satisfaction data as a critical component of performance improvement and clinical effectiveness programs are required [7, 8].

Patient satisfaction, which is a perception and an attitude that a consumer can have or view towards a total experience of health care, is a multidimensional aspect, which represents a vital key marker for the quality of health care delivery [1, 2, 4, 9]. Furthermore, level of patient satisfaction is an internationally accepted factor, which needs to be studied routinely to complement other methods of quality assessment and assurance for smooth functioning of the health care system [2, 3, 5, 9–11]. This wholistic approach can help to better meet patients' needs and improve quality of health service delivery by identifying and understanding of its determinants through a continuous quality improvement process [3, 10, 12].

Various dimensions of patient satisfaction have been identified, health care services ranging from admission to discharge process, waiting time to receive care, as well as from medical care to interpersonal communication [13–16]. It has also been reported that the interpersonal and technical skills of health care provider are the two unique dimensions involved in a patient assessment of hospital care [17–20]. General amenities of the facility in terms of availability, its quality, and structural design were also among the identified dimensions, which significantly affect the patient satisfaction level [2, 21, 22].

Additionally, sociodemographic and economic status of the patients and their expectations of care and attitudes towards the health care system were among the dimensions identified to have direct influence on satisfaction level; other psychosocial factors, including pain and depression, are also known to contribute to patient satisfaction level scores [9, 23–29].

In Ethiopia, the health care services are limited and of poor quality, which is a direct reflection of the countries socioeconomic status. This takes the lion share for the higher morbidity and mortality of the population due to the emerging and reemerging infectious and chronic health problems in the nation. The lower life expectancy rate of the peoples in the nation is a clear indication for this.

Considering this major bottleneck for the health system, the Ethiopian government has focused on improving the health care organizations and quality of health service delivery to the population in recent years. In such efforts towards improving the quality health care service delivery, measuring the level of patient satisfaction is an integral component of the health system for continuous quality improvement process [30]. On top of this, following increased levels of competition and the emphasis on consumerism, measuring patient satisfaction has become an important measurement for monitoring health care performance of health plans.

Therefore, this study is aimed at measuring the level of patient satisfaction and its determinants in one of the specialized hospitals in Addis Ababa so as to identify the critical dimensions, which has a greater influence on patient satisfaction level related to inpatient services in Ethiopian context.

### 1.2. Justification of the Study

In the context of Ethiopian health sector reform, health facilities are striving to improve the efficiency, effectiveness, and quality of services provided. Among the range of indicators that has been used to measure quality health service delivery and to track its change over time in Ethiopia, routine measuring of patient satisfaction level for improvement is the recommended approach for health care providers at all level [30]. On top of this, improving the patient's satisfaction level by identifying the major responsible factors for improvement is the integral component of continuous quality improvement process and efficient way.

It is generally agreed that satisfaction data's play a significant role in improving the strategy and tactics of health care providers in delivering health care services for clients. In addition to its role in improving quality health service delivery, measurement of patient satisfaction plays an important role in the growing push toward accountability among health care providers, which has a significant role in developing and delivering high-quality health care in the hospital with the involvement of patients in the management of their problem and treatment [9, 11, 31]. It is also viewed as an established indicator for quality of care, despite it was overshadowed by measures of organizational aspects in the quality of health care equation [30].

Different studies also indicated that a satisfied patient has complied with the medical treatment prescribed, service provider recommendation, and continually using medical services at a specific health provider, which might result with the enhanced disease healing process and healthier and happier clients, who are contributing to the development of the country [14, 32].

## 2. Methods

### 2.1. Study Setting, Sample Size, and Sampling Procedures

This study was conducted at one of the specialized governmental hospitals in Addis Ababa, Ethiopia, aimed at assessing the level of patient satisfaction and its determinants. A facility-based cross-sectional study design was applied to understand patient's satisfaction level by the services provided in the facility. All admitted patients at Black Lion Specialized Hospital during the study period were used as source population for this study. All admitted patients at different wards who stayed for 48 hrs and above during the time of data collection were the study population. During the data collection, patients who were selected and fulfilled the inclusion criteria for the study among the admitted patients were included in the study. Those seriously ill, laboring mothers, and pediatric patients without parents/guardian were excluded from the study.

The total sample size of this study was determined using a single population proportion formula by taking the assumptions that the overall net patient satisfaction to be 62% [33], 95% CI and a 5% margin of error (d), and expected a nonresponse rate of 10%. This resulted in a total of 398 samples for the study. Available bed capacity of wards and available patients during the study period was used to allocate the total sample size in each ward to ensure the proportional allocation of samples from all service providing ward of the hospital. Health Management Information System (HMIS) report of the hospital made on October 2015 was used to know the total available beds per ward and estimate the patients per ward. Systematic random sampling technique was used to ensure the random selection of participants with every three intervals to recruit study participants. Lists of patients from the patient register of an individual ward were used as the sampling frame for selection.

### 2.2. Study Variables

#### 2.2.1. Dependent Variables

Level of patient satisfaction with inpatient service was considered the dependent variable.

#### 2.2.2. Independent Variables

(i) Sociodemographic variables
    Sociodemographic variables including sex, age, marital status, living with status of the study participant, educational status, occupational status, religion, ethnicity, permanent residence of the patient, and financial income status of the respondents were considered. Besides, presence of caretaker during hospital admission was also considered.
(ii) Service utilization-related information variables
    Information regarding waiting time to get required service in the facility (as stated by the participant), official visiting hours of the facility, information's received regarding the services provided by the hospital, admitting processes of the hospital for inpatient service, activities performed to ensure privacy of the patient, measures taken to assure confidentiality, and availability of signboards displayed inside the hospital were collected.
(iii) Patient and health care provider interaction variables
    Health education provided by health workers, nursing care received during their stay, perceived quality of physician service received, quality of inpatient pharmacy service, abundancy on supply and availability of drugs in the hospital, laboratory and radiology services of the hospital, and the hospital pain management were considered.
(iv) General facility amenities of the hospital-related variables
    Under this category, availability of rooms for accommodation, dietary service given by the hospital, toilet, ward and bed services, and cleanliness were considered.

### 2.3. Data Collection Materials and Procedures

A structured questionnaire developed by reviewing other similar studies [12, 33–38] was used for questionnaire development, which we used to assess the level of patient satisfaction with inpatient services in Black Lion Specialized Hospital. To ensure the required information is collected with greater understanding, local language was used during data collection. To facilitate this, the data collection questionnaire was first developed in English, then translated to Amharic language, and back to English for its consistency. Three diploma level health professionals were used for data collection, and one first degree level health professional with three years of experience and one of the principal investigators acted as a supervisor during the data collection. The data collection team was briefed about the objective and methodology of the study and trained on the data collection process by the principal investigators. To avoid social desirability bias, both the data collectors and supervisors were recruited from other health facilities. Admitted patients were interviewed being on their bed. To ensure the quality of the collected data, the collected data have been collected by the supervisors to review for clarity and completeness check on a daily basis.

### 2.4. Measurements

#### 2.4.1. Patient Satisfaction

Considering that patient satisfaction is a collective outcome of different kinds of services provided in the hospital, the level of patient satisfaction in this study was measured by using 19-item questions, which is composed of three dimensions. Service utilization, patient and health care provider interaction, and facility-related information were the three different dimensions assessed. Each item has a 5-point Likert scale ranging from 1 (very dissatisfied) to 5 (very satisfied).

#### 2.4.2. Overall Patient Satisfaction

This is measured by using one item in the questionnaire stating “How do you rate your overall level of satisfaction regarding the health service you received in this hospital?”

#### 2.4.3. Net Overall Patient Satisfaction

Mean value of participants' level of satisfaction which was computed from the entire questions under the three dimensions (service utilization, patient and health care provider interaction, and facility-related information) was used (Table 1).

### 2.5. Data Processing and Management

The collected data were entered and analyzed using SPSS version 21 statistical software. After data cleaning was completed, descriptive statistics including frequencies and percentages were used to describe the study participants. Before fitting to the model for analysis, required assumption for the model in use was done. Statistical tests for normality such as Shapiro–will and Kolmogorov–Smirnov were performed. During checking for the assumption for linear regression, the dependent variable was not normally distributed, and a two-step transformation was performed to ensure its normality. To reduce the load of the independent variables and select critical variables, factor analysis with varimax rotation was performed, variables with greater than 0.443 rotated component matrix value were considered, and bivariate analysis was performed for every independent variable with the outcome variable independently. Finally, explanatory variables which had a statistically significant association with the dependent variables at *p* < 0.05 were considered for the final linear regression model.

### 2.6. Data Quality Management

Quality of the data for the study was assured throughout the study period starting from the designing phase of a data collection instrument. Simplicity of the questions for understanding and relevance of variables in the study were considered during preparation followed pretesting, and modification of the questionnaire based on the findings was performed accordingly. Before actual data collection, training was given for data collectors and the supervisor on the techniques of data collection, proper categorization, and coding of data. During data collection, the assigned supervisor and principal investigator checked the collected data on a daily basis for its completeness, accuracy, and clarity. Cronbach alpha was calculated for all independent variables together in order to demonstrate the reliability of the variables to measure the overall satisfaction level of the patients during analysis stage. The Cronbach alpha analysis indicated that reliability for variables included in the study was 0.81.

### 2.7. Ethical Considerations

Ethical approval was obtained from the Ethical Review Committee of Jimma University, College of Health Sciences. Verbal consent was also obtained from the study participants before the data collection after providing clear information on the study objective and other methodological issues. During the data collection, names of the participants were kept anonymous by using a study record number only. It is believed that there is no anticipated harm for the patients except for their time scarification at the time of data collection.

## 3. Results

### 3.1. Sociodemographic Characteristics

A total of 398 patients were participated in the study making a response rate of 100%. Of the total study participants, 231 (58.0%) and 211 (53.0%) were female and married, respectively. The median age of study participants was 34 years. One hundred twenty (30.2%) study participants had primary education and 69 (17.3%) of them have no formal education. The median income of the study participants was 1,500.00 Ethiopian Birr (ETB), and 273 (68.6%) of the participants were from a rural part of the country (Table 2).

### 3.2. Hospitalization Characteristics

About 249 (62.6%) study participants were admitted for the first time to the hospital, and 222 (55.8%) of the respondents were paying for the hospital service. Among study participants, 239 (60.1%) of them stayed in the hospital for less than 15 days (Table 3).

### 3.3. Level of Patient Satisfaction with Different Service Categories

Among the three service categories used to assess the net overall satisfaction rate of patients, the majority of the patients were satisfied with the services under patient and health care provider interaction and facility-related information. This study finding showed that almost half of the study participants were satisfied with waiting time to get service 190 (47%), 228 (57.3%) with official visiting hours of the hospital, 207 (52.0%) with information provided on the available service by the staffs, 194 (48.7%) with admission processes of the hospital, 169 (42.5%) and 219 (55.0%) with measures taken to assure confidentiality and privacy of the patients, respectively, and 187 (47.0%) with signboards available inside the hospital (Table 4).

In patient and health care provider interaction, a total of 235 (59.0%) patients claimed that they were satisfied with the physician service. The highest very dissatisfaction proportion in this study was registered regarding the observed cleanness status of the toilets in the facility. A total of 42 (10.6%) study participants claimed that they were very dissatisfied with the cleanness of toilets in the facility (Table 4).

### 3.4. Overall Net Patient Satisfaction Level

**Figure 1:**
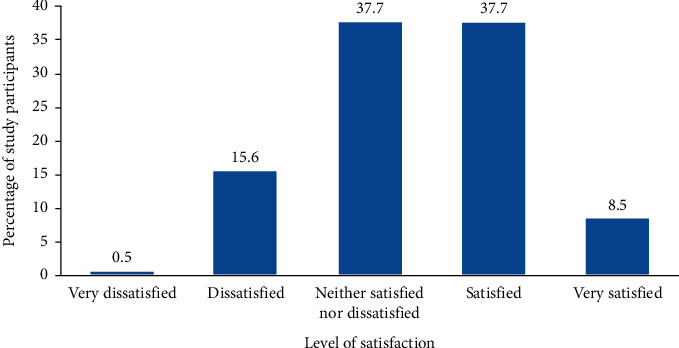
Level of overall net patient satisfaction of study participant of Black Lion Specialized Hospital (BLSH), December 2015.

Regarding the overall satisfaction level with the hospital service, around 150 (37.7%) patients were satisfied with the service provided by the hospital, and an equivalent number of study participants were neither satisfied nor dissatisfied. Besides, 8.5% of the respondents were very satisfied with the service, and around 0.5% of the respondents were very dissatisfied with the service provided by the hospital service (Figure 1).

### 3.5. Patients Satisfaction by Sociodemographic and Hospitalization Variables

In this study, the sociodemographic and hospitalization characteristics variables were found to explain 27.1% of the variability in the net overall satisfaction score. Among the sociodemographic and hospitalization characteristics measuring variables to measure the net overall satisfaction level, patient's age, marital status, and presence of caretaker during admission shows independent association with net overall satisfaction level at *p* < 0.05. The net satisfaction level of study participants aged >34 increased by 3.45 (95% CI: −1.33 to 5.55) as age increased by one unit. The net satisfaction score of study participants who have their own caretaker in their hospital stay was increased by 4.66 (95% CI: −1.05 to 8.27) when compared with study participants without a caretaker in their hospital stay.

### 3.6. Patient's Satisfaction by Service Utilization-Related Information, Patient-Health Care Provider Interaction, and Facility-Related Information Variables

The relationship between service utilization-related variables, patient and health care provider interaction variables, and facility-related information variables with net overall satisfaction is quantified in Table 5. These variables were found to explain 96.5% of the variability in the net overall satisfaction score. For almost all variables in these domains, when facility service improves, the regression estimates of satisfaction level moved from very dissatisfied to very satisfied, which means that an improvement of listed services to satisfy the clients can potentially have an improvement in the net satisfaction score of patients.

### 3.7. Final Predictors of Patient Satisfaction in Black Lion Hospital

The variables included in the final model explained 96.4% of the variability in net overall patient satisfaction. Physician service, laboratory and radiology services, pain management, inpatient pharmacy service, toilet cleanliness, room accommodation, and dietary service were strong predictors of patient satisfaction.  Table 6 provides the regression estimates and the relative effect of each predictor variable for net overall patient satisfaction with all variables, which were included for the final multilinear regression model.

From the variables categorized into three parts, none of the variables under service utilization-related indicators were predictors of net overall satisfaction. Majority of variables under patient and health care provider interaction variables (physician service, laboratory and radiology services, pain management, and inpatient pharmacy service) were significant predictors of net overall satisfaction of patients.

As it is indicated in the table, a unit increases in physician service score will lead to 1.423 unit times increase in the net overall satisfaction score with a 95% CI of 1.164–1.682 at a *p* value less than 0.001. Pain management for patients was also a strong predictor of patient satisfaction. In a unit, increase in pain management score will lead to 1.379 increases in the patient satisfaction score with a 95% CI of 1.141–1.617 at a *p* value of less than 0.001. From all predictor variables, inpatient pharmacy services have a significant change in the satisfaction score of patients in BLSH. A unit increase in the inpatient pharmacy will increase the satisfaction score of patients by 2.311 unit with a 95% CI of 2.118–2.504 at a *p* value of less than 0.001.

From the third category (i.e., facility amenity-related variables), three variables (toilet cleanliness, room accommodation, and dietary service) were significant predictors of net overall satisfaction of patients in BLSH. From these variables, toilet cleanliness was a strong predictor variable of net overall satisfaction score. A unit improvement in toilet cleanliness will increase the satisfaction level of patients by 1.500 unit with a 95% CI of 1.300–1.700 at a *p* value of less than 0.001. Room accommodation had also a significant change in net overall satisfaction score of study participants. The net overall satisfaction score of study participants increased by 1.311 unit when there is a unit improvement in room accommodation with a 95% CI of 1.300–1.700 at a *p* value of less than 0.001. Similarly, a unit improvement in dietary service showed that 1.211 unit satisfaction level improvement with a 95% CI of 1.008–1.413 at a *p* value of less than 0.001 (Table 6).

## 4. Discussion

Our study finding shows that the proportion of overall net patient satisfaction rate is 46.2%. This level of satisfaction was higher when compared with other interventional study performed in Debre Markos Hospital, Ethiopia, which is 25% before the intervention [39]. But, compared with other studies performed on patient satisfaction in Jimma Specialized Hospital, Debre Berhan Hospital, and other study performed in Bahir Dar Felege Hiwot Hospital, the overall satisfaction level was low, which is 61.9%, 57.7%, and 57.8%, respectively [33–36]. This discrepancy may be due to the level of the hospital and/or workers capacity and motivation status of the health workers to attain higher patients' needs.

Different study findings revealed that the relevance of information services given for patients, proper admitting processes, short waiting time to receive service, and ensuring privacy and confidentiality for services provided by the hospital had statistical association in other studies. On the other hand, different literatures also indicate that quality of communication and interpersonal skills is also the key indicator for patient satisfaction [13–20]. But none of the variables were found to be significant for patient satisfaction in our study. This may be due to the difference in the type and level of hospital, patients flow, patient-level expectations, and priority of required services of the patients. Black Lion Hospital is one of the specialized hospitals in the nation, which is responsible to receive referral patients from all over the nation, and the type of conditions to be managed in this hospital is very serious, which needs specialized service. Besides, there are many services which are found in this hospital only. Considering these, clients may prioritize receiving care rather than the amount of time they spent to receive. So that, the clients who are coming to this facility may expect to wait longer time duration and compromised other facility-related services. This trend may be perceived as normal compared with the quality of clinical services they want to receive, which may be their priority as it is indicated in our finding that a total of 180 (45.2%) participants wanted improvement in laboratory and pharmacy services.

Majority of variables under patient and health care provider interaction domain (physician service, laboratory and radiology services, pain management, and inpatient pharmacy service) were significant predictors of net overall satisfaction of patients. From this domain, inpatient pharmacy service was the strong predictor variable of net overall satisfaction score of the patients.

The net overall satisfaction of study participants satisfied with physician service in the facility had 1.423 unit times greater satisfaction score with the service (95% CI (1.164–1.682)) at *p* < 0.001. A unit increase in the quality of laboratory and radiology services score will lead to 0.706 unit times increase in net overall satisfaction level of patients with a 95% CI of 0.478–0.935 at a *p* value of less than 0.001. Our study finding is also in line with other studies performed in other different areas [33, 37, 40]. Pain management for patients is also strong predictors of patient satisfaction in our study, which is against the study finding performed in other study areas [28]. Based on this study finding, the net overall satisfaction score of patients increases by 1.379 when there is a unit increase in the pain management with a 95% CI of 1.141–1.617 at *p* < 0.001.

Regarding inpatient service of the facility, about half of (52.5%) of the participants were satisfied with the service they received in the hospital. This finding is lower than the study performed in India on patient satisfaction in a tertiary care teaching hospital, which shows about 69% of respondents were satisfied by the pharmacy service they received from the hospital. The possible explanation for this difference may be due to the difference in developmental status of the two countries. India is more developed country than Ethiopia and might have a strong health system structure with numerous pharmaceutical factories, which may be the reason for the abundant availability of drugs in the facility that can satisfy the patients need. But it is almost similar with other study performed in Jimma Specialized Hospital, in which 54% of patients were satisfied by the drug availability and supply in the inpatient pharmacy.

Inpatient pharmacy service is found to be the highest predictor of patient satisfaction in our study. The finding shows that a unit increase in the inpatient pharmacy services increases patient satisfaction level by 2.311 unit with a 95% CI of 2.118–2.504 at *p* < 0.001. Another prior study conducted in a similar facility (Black Lion Hospital) before four years also revealed that the main reasons for patient's dissatisfaction in the hospital was the poor quality services they received from pharmacy, radiology, and laboratory department of the hospital, which can significantly indicate the persistence of the problem in the facility after four years [12, 33–35, 38, 41, 42].

From the general facility amenity-related domain, three variables (toilet cleanliness, room accommodation, and dietary service) were significant predictors of net overall satisfaction of patients in BLSH. From these variables, toilet cleanliness was a strong predictor variable of net overall satisfaction score of the patients.

Based on this study finding, a unit improvement in toilet cleanliness will have an average of 1.500 times increment in the net overall satisfaction rate of patients in the hospital with 95% CI (1.300–1.700) at a *p* value of less than 0.001 and a unit increase in dietary services score also increase patient satisfaction by 1.211 unit with 95% CI (1.008–1.413) at a *p* value of less than 0.001. Other study findings in other areas also showed that dietary service, cleanness of toilet and bed were an important predictor of patient satisfaction [33, 34, 36, 40, 42–45]. This result can be supported by the finding that, the highest “very dissatisfaction” level among patients (10.6%) were with toilet cleanness problem. Almost 50% of participants were dissatisfied with the toilet service of the hospital. Our study finding is also similar with the study done in Bangladesh. Which indicate that a common complaint of patients was against cleaner and sweepers of the hospital that they do not clean the hospital toilet and bathroom regularly [34]. The possible reason for their complaint from patients' sides maybe, they believe that receiving adequate food timely, clean toilet service and good quality accommodation are the bases for psychological satisfaction and healing process. Besides, they may fear to develop health care -acquired infection due to the poor sanitation of the facility which can debilitate their health status.

In this study, none of the sociodemographic characteristics of the patients were found to be a predictor for the net overall satisfaction of the patients. Other similar studies on the influence of sociodemographic characteristics on patients' satisfaction also showed that there is no consistent relationship between patient satisfaction with age, race, gender, education, or income. However, some other studies found that older patients were more likely to report satisfaction compared with younger patients and that females were more likely to express satisfaction than males [26, 27, 35].

In general, this study found that patient and health care provider interaction domain and general facility amenity-related domains of patient satisfaction were found to explain 96.4% of the variability in the net overall satisfaction score of the patients. Another new domain dimension of patient satisfaction including facility design domain should be considered for future research.

## 5. Recommendations

Continuous quality improvement is linked to the use of timely and useful feedback from clients. Patients constitute the hospital's direct client. The overall satisfaction is an important aspect of the service itself, and it is considered to be an important outcome measure for health services. Patient care is not considered to be of high quality unless the patient is satisfied.

Improving patient satisfaction is one of the key indicators of quality of care and indicator of quality health service. Based on this study finding, we recommend the following strategies to improve patient satisfaction in the hospital.

- The hospital managers should routinely assess the patients' satisfaction status and provide tailored on-the-job training to their professionals in the facility in order to improve their skill for enhancing patients' satisfaction.
- Managers of the hospital should devise a mechanism to improve the performance of staff and monitor the implementation process in order to take appropriate improvement and action.
- Periodic assessment of health services and further study in other dimensions of patient satisfactions including facility design dimension, which can explore from the user's satisfaction perspective, is recommended as a fundamental initiative in the improvement of the performance of health care delivery in the facility.
- Hospital reformation and modern hospital administration system could be considered to improve the level of patient satisfaction.
- The hospital should improve the general facility amenities including the meal need of the patient and the cleanliness of the toilet.
- Considering that service unavailability is a major source of patient dissatisfaction, a system should be in place to ensure the availability of hospital-specific tracer drugs in the hospital on a daily basis, and there should be drug information service in the hospitals as recommended by Ethiopian Hospital Reform Implementation Guideline.
- Improving the client-patient interaction should be addressed by using an appropriately designed checklist which considers the norm of the community and other client needs, and a standard checklist for communication can potentially improve the satisfaction level of patients regarding service delivery of different departments.
- Improving the health literacy status of service providers in the facility should be addressed by providing on-the-job trainings related to determinants of patients as soon as possible to support the compassionate and respectful care initiative of the hospital.
- Last, we want to emphasize that this research identifies a number of indicators that are important to improve the quality of hospital services and patient satisfaction in Black Lion Specialized Hospital. Since the study is conducted in one government medical hospital in Addis Ababa, we caution against generalizing the results to the context of the entire country. But other hospital authorities may consider the tool and findings of this study to improve the services of the hospital for patient satisfaction and better treatment.

## 6. Conclusion

Based on the study finding, around 46.2% of the patients were satisfied by the services they received in the hospitals, and 15.6% of the patients reported that they were dissatisfied by the hospital services. The remaining 37.7% of the respondents were neither satisfied nor dissatisfied with the service provided in the hospital service. Based on this finding, the overall patient satisfaction is low compared with other health facilities in the nation and compared with the average standard level expected. Majority of variables under patient and health care provider interaction variables and facility amenities have higher prediction capacity for the net overall satisfaction of patients. Physician service, laboratory and radiology services, pain management, inpatient pharmacy service, cleanliness of the toilet, room accommodation, and dietary service of the hospital were significant predictors of net overall satisfaction of patients in Black Lion Specialized Hospital.

## Figures and Tables

**Table 1:** Operational definitions used.

S. no	Variable	Definition used
(1)	Patient satisfaction	Perception of the patients regarding the outcome of care and the extent it meets their needs and expectations
(2)	Inpatients	Patients who had a hospital stay greater than 48 hours in the facility
(3)	Service departments	Service area serving inpatients in the facility during their stay in the hospital
(4)	Waiting time	The time interval between the departure from registration for inpatient service and seen by a doctor to receive service

**Table 2:** Sociodemographic characteristics of study participants in Black Lion Specialized Hospital (BLSH), Addis Ababa, Ethiopia, December 2015 (*N*  = 398).

Variables	Categories	Frequency	Percentage (%)
Sex	Male	167	42.0
	Female	231	58.0

Age	≤34 years	199	50.0
	>34 years	199	50.0

Marital status	Single	128	32.2
	Married	211	53.0
	Divorced	34	8.5
	Widowed	25	6.3

Presence of caretaker	Yes	361	90.7
	No	37	9.3

Educational status	No formal education	69	17.3
	Elementary/1–8th grade	120	30.2
	Secondary school	95	23.9
	Vocational/diploma	66	16.6
	Degree and above	48	12.1

Occupational status	Government employee	83	20.9
	Private/NGO	132	33.2
	Farmer	56	14.1
	Student	48	12.1
	Daily laborers	16	4.0
	Merchants	54	13.6
	Others	9	2.3

Religion	Orthodox	224	56.3
	Protestant	82	20.6
	Muslim	86	21.6
	Others	6	1.5

Ethnicity	Amhara	150	37.7
	Oromo	142	35.7
	Tigre	46	11.6
	Gurage	49	12.3
	Other	11	2.8

The region they come	Addis Ababa	124	31.2
	Amhara	70	17.6
	Oromiya	124	31.2
	Tigray	35	8.8
	SNNP	38	9.5
	Others	7	1.8

Residence	Urban	273	68.6
	Rural	125	31.4

Financial income status of the respondents	Less than 1000ETB	152	38.1
	1000–2500ETB	173	43.5
	2500–5000ETB	71	17.8
	Greater than 5000ETB	2	0.6

**Table 3:** Hospitalization characteristics of study participants in Black Lion Specialized Hospital (BLSH), Addis Ababa, Ethiopia, December 2015 (*N*  = 398).

Variables	Categories	Frequency	%
Number of days of hospital stay	<7 days	140	35.2
	7–14 days	99	24.9
	14–21 days	79	19.8
	21–28 days	25	6.3
	28–35 days	26	6.5
	35–42 days	11	2.8
	42–49 days	5	1.3
	49–56 days	1	0.3
	>56 days	12	3.0

Admission type	New	249	62.6
	Repeat	149	37.4

Payment status	Free	176	44.2
	Paying	222	55.8

Ward	Medical ward	75	18.8
	Surgical ward	56	14.1
	Gynecology and obstetrics	88	22.1
	Orthopedics	27	6.8
	Pediatrics	85	21.4
	Oncology	67	16.8

**Table 4:** Satisfaction levels of study participants in Black Lion Specialized Hospital (BLSH) with different service categories, Addis Ababa, Ethiopia, December 2015 (*N*  = 398).

Variables	Very dissatisfied	Dissatisfied	Neutral	Satisfied	Very satisfied
*Services utilization-related information*					
Waiting time to get service	32 (8.0%)	114 (28.6%)	26 (6.5%)	190 (47.7%)	36 (9.0%)
Visiting hours	8 (2.0%)	61 (15.3%)	36 (9.0%)	228 (57.3%)	65 (16.3%)
Information on the service	21 (5.3%)	99 (24.9%)	48 (12.1%)	207 (52.0%)	23 (5.8%)
Admission processes	27 (6.8%)	108 (27.1%)	43 (10.8%)	194 (48.7%)	26 (6.5%)
Privacy	19 (4.8%)	63 (15.8%)	38 (9.5%)	219 (55.0%)	59 (14.8%)
Measures taken to assure confidentiality	26 (6.5%)	124 (31.2%)	42 (10.6%)	169 (42.5%)	37 (9.3%)
Signboards	27 (6.8%)	105 (26.4%)	40 (10.1%)	187 (47.0%)	39 (9.8%)

*Patient and health care provider interaction*					
Health education provided by the health workers	23 (5.8%)	140 (35.2%)	42 (10.6%)	165 (41.5%)	28 (7.0%)
Nursing care	14 (3.5%)	47 (11.8%)	29 (7.3%)	242 (60.8%)	66 (16.6%)
Physician service	11 (2.8%)	39 (9.8%)	33 (8.3%)	235 (59.0%)	80 (20.1%)
Inpatient pharmacy services	25 (6.3%)	126 (31.7%)	38 (9.5%)	175 (44.0%)	34 (8.5%)
Availability of drug and supply	10 (2.5%)	67 (16.8%)	22 (5.5%)	236 (59.3%)	63 (15.8%)
Laboratory and radiology services	25 (6.3%)	72 (18.1%)	43 (10.8%)	216 (54.3%)	42 (10.6%)
Pain management	20 (5.0%)	65 (16.3%)	30 (7.5%)	224 (56.3%)	59 (14.8%)

*Facility amenity-related information*					
Room accommodation	10 (2.5%)	67 (16.8%)	22 (5.5%)	236 (59.3%)	63 (15.8%)
Dietary service given by the hospital	28 (7.0%)	79 (19.8%)	45 (11.3%)	205 (51.5%)	41 (10.3%)
Toilet cleanliness	42 (10.6%)	124 (31.2%)	33 (8.3%)	164 (41.2%)	35 (8.8%)
Ward cleanliness	8 (2.0%)	51 (12.8%)	57 (14.3%)	225 (56.5%)	57 (14.3%)
Bed cleanliness	6 (1.5%)	45 (11.3%)	30 (7.5%)	242 (60.8%)	75 (18.8%)

**Table 5:** Relationship of net overall patient satisfaction level with services of BLSH, Addis Ababa, Ethiopia, December 2015.

Variables	B	95% CI		*p* value
		Lower B	Upper B	
*Services utilization-related information*				
Information on services provided	5.957	5.157	6.757	<0.001
Admitting processes of the hospital	5.213	4.419	6.006	<0.001
Waiting time to receive service	1.891	1.016	2.766	<0.001
Official visiting hours for service	2.814	1.773	3.855	<0.001
Available signboards in the facility	5.313	4.558	6.068	<0.001

*Patient and health care provider interaction*				
Nursing care	4.664	3.691	5.637	<0.001
Physician service	5.561	4.590	6.533	<0.001
Health education	2.834	1.937	3.732	<0.001
Laboratory and radiology services	5.478	4.676	6.280	<0.001
Pain management	5.576	4.765	6.387	<0.001
Inpatient pharmacy services	5.084	4.322	5.846	<0.001

*Facility-related information*				
Toilet cleanliness	5.292	4.601	5.984	<0.001
Ward cleanliness	6.942	6.033	7.850	<0.001
Room accommodation	6.565	5.741	7.390	<0.001
Bed cleanliness	7.375	6.467	8.282	<0.001
Dietary service	1.861	0.938	2.784	<0.001

**Table 6:** Final predictor variables of net overall patient satisfaction in BLSH, Addis Ababa, Ethiopia, December 2015 (*N*   = 398).

Variables (categories)	Unstandardized B-coefficient	95% confidence interval		*p* value
		Lower B	Upper B	
*Age*				
≤35.5 years	—			
>35.5 years	0.05			0.662

*Presence of caretaker*				
No	—			
Yes	0.00			0.975

*Marital status*				
Single	0.141	0.079	1.625	
Married	0.091	0.072	1.253	
Divorced	0.034	0.015	1.139	
Widowed	0.044	0.021	1.027	

*Services utilization-related information*				
Information services	0.902	0.653	1.152	
Admitting processes	1.147	0.922	1.373	
Waiting time	0.978	0.786	1.169	
Visiting hours	0.872	0.608	1.135	
Signboards	1.039	0.815	1.263	

*Patient and health care provider interaction*				
Nursing care	1.157	0.919	1.395	
Physician service	1.423	1.164	1.682	<0.001^*∗∗*^
Health education	1.006	0.811	1.201	
Laboratory and radiology services	0.706	0.478	0.935	<0.001^*∗∗*^
Pain management	1.379	1.141	1.617	<0.001^*∗∗*^
Inpatient pharmacy services	2.311	2.118	2.504	<0.001^*∗∗*^

*Facility-related information*				
Toilet cleanliness	1.500	1.300	1.700	<0.001^*∗∗*^
Ward cleanliness	1.233	0.946	1.52	
Room accommodation	1.311	1.043	1.58	<0.001^*∗∗*^
Bed cleanliness	1.225	0.92	1.529	
Dietary service	1.211	1.008	1.413	<0.001^*∗∗*^

^*∗∗*^Variables having *p* value <0.001 and found to be strong predictors of the final model.

## Data Availability

The data used to support the study are available from the corresponding author upon request.
